# Brain Volumes and Metacognitive Deficits in Knowledge of Self, Task and Strategies in Mathematics: A Preliminary Pilot One-Year Longitudinal Study in aMCI Patients Compared to Healthy Controls

**DOI:** 10.3390/diagnostics13040680

**Published:** 2023-02-11

**Authors:** Vaitsa Giannouli, Magdalini Tsolaki

**Affiliations:** 1 School of Medicine, Aristotle University of Thessaloniki, 54124 Thessaloniki, Greece; 2 Department of Psychology, University of Western Macedonia, 53100 Florina, Greece

**Keywords:** metacognitive deficts, mathematics, metacognitive avoidance strategies, brain volumes, amygdala, aMCI, healthy controls

## Abstract

Metacognitive knowledge has been little investigated in aMCI patients. The aim of this study is to examine whether there are specific deficits in knowledge of self, task and strategies in mathematical cognition, due its importance for everyday functioning, mainly due to its importance for financial capacity in old age. A total of 24 patients with a diagnosis of aMCI and one-to-one 24 matched individuals (similar age, education and gender) were examined at three time points in a year with a number of neuropsychological tests and a slightly modified version of the Metacognitive Knowledge in Mathematics Questionnaire (MKMQ). We analyzed longitudinal MRI data regarding various brain areas for the aMCI patients. Results indicated that the aMCI group differed in all MKMQ subscale scores at the three time points compared to healthy controls. Correlations were found only for metacognitive avoidance strategies and left and right amygdala volumes at baseline, while after twelve months correlations were found for avoidance and right and left parahippocampal volumes. These preliminary results highlight the role of specific brain regions that could be used as indices in clinical practice for the detection of metacognitive knowledge deficits that are found in aMCI.

## 1. Introduction

According to Flavell [1], metacognition refers to the awareness that a person has about their knowledge as well as the regulation of processes involved in learning in order to meet the demands of specific tasks; thus, metacognition includes metacognitive knowledge (MK) and metacognitive experiences (ME) or regulation. More specifically, metacognitive knowledge encompasses knowledge of person variables, task variables and strategy components, and can be defined as the declarative knowledge that a person has regarding their own personal experiences with specific tasks (e.g., mathematical), along with theories and relevant beliefs about cognition and thinking [1]. A plethora of published studies supports the idea that patients diagnosed with varying severity of neurocognitive disorders due to different etiologies demonstrate changes in MK [2,3]. More specifically, in older adults, metacognitive control is not severely affected, while metacognitive awareness/beliefs about individual skills show a pattern of decline [4].

Prior research in Greece in patients with a diagnosis of mild cognitive impairment (MCI)/mild neurocognitive disorder shows that they overestimate their performance in every cognitive domain, in comparison with healthy controls who underestimate their performance in measures of verbal memory [5]. Another study in Greek older patients extends these findings and supports the idea that mild Alzheimer’s disease (AD) patients show the highest overestimations of their cognitive skills in different domains, followed by single and multiple domain amnestic MCI (aMCI) and healthy older controls [6]. This impairment in metacognition taking the form of self-awareness deficits has also been supported by other researchers, who support a prominent deficit in self-awareness in MCI, but not as prominent as in the case of AD patients [7,8,9]. Nevertheless, a meta-analysis indicates that MCI patients have knowledge of their cognitive deficits, and that the level of awareness seems to vary according to three predictors, namely, cognitive status, language and memory abilities [10]. Therefore, there are attempts to quantify the exact cut-off scores that can be used for Mini Mental State Examination (MMSE) in order to predict insight or awareness of deficits, with high MMSE scores (≥24) correlating with higher metacognitive awareness, and a noticeable decrease found for scores MMSE (=23 to 13), while low metacognitive knowledge is found for MMSE scores (≤12) [11].

Financial capacity is considered to be a broad and complex psychological construct, that some researchers approach as one of the Instrumental Activities of Daily Living (IADLs), which is of extreme importance for older populations. It is worth mentioning that knowledge of skills relating to mathematics is involved in the design of financial capacity instruments that are in use in neuropsychological assessment in different cultural settings and, according to a body of studies, relevant deficits can be found in (Greek) older patients with vascular dementia (VD) [12], AD [13], and Parkinson’s disease with dementia (PDD) [14]; similar findings have also been reported for aMCI patients [15].

Mathematical/financial problems are part of the instruments measuring financial capacity [16] and have shown that patients suffering from fronto-temporal dementia (FTD) overestimate their performance [17], a finding that is also detected as distorted self-awareness (overestimations) in MCI patients, as well as in mild and moderate AD patients, Parkinson’s disease patients [18], and in patients with dementia with Lewy Bodies [19].

An interesting association has been found between medial prefrontal and anterior temporal cortices’ decreased activity and impaired self-awareness in AD patients [20], while inaccurate self-evaluations of cognitive domains, such as memory, seems to be controlled by the prefrontal cortex [21]. Of relevant interest is also the atrophy in frontal and parietal lobes [22] and more specifically of the angular gyrus in the left parietal lobe [23] in amnestic MCI (aMCI) [24] as well as in mild AD patients [25].

Although questionnaires have been used in prior research to measure general metacognitive awareness, so far notions such as metacognitive knowledge of the self (easiness, fluency) (e.g., statements like ‘I solve mathematical problems easily no matter how many operations they require’), metacognitive knowledge of the self (difficulty, lack of fluency) (e.g., ‘When I solve problems with multiplications I get tired’), metacognitive knowledge of tasks (easy, low demands) (e.g., ‘I find the problems that require subtraction difficult’), metacognitive knowledge of tasks (difficult, high demands) (e.g., ‘I think that a problem that has fractions is difficult’), metacognitive knowledge of strategies (cognitive/metacognitive strategies) (e.g., ‘When I am reading a mathematical problem I am thinking whether there are various ways of solving it’), metacognitive knowledge of strategies (competence/enhancing strategies) (e.g., ‘When I solve mathematical problems I am thinking of other similar ones from everyday life’), and metacognitive knowledge of strategies (avoidance strategies) (e.g., ‘When the mathematical problem is difficult I give up’) that were introduced by Efklides and Vlachopoulos [26] have not been systematically investigated in aMCI patients, and the biological substrate of such constructs is still unknown. So far, no specific instrument has been used for the measurement of metacognitive knowledge regarding self, task and strategies in mathematics (except for items coming from financial capacity instruments) in patients suffering from neurocognitive disorder.

The aim of this study is to explore for the first time metacognitive knowledge in mathematics at multiple time points and to provide not only evidence from a longitudinal perspective regarding aMCI patients, but also to examine whether there are specific brain regions where neuronal death and change of volume may be used as future indices/predictors of impairment. Therefore, the research questions that were examined in this study are: (a) Do specific self-reported metacognitive knowledge aspects, such as metacognitive knowledge of the self (difficulty, lack of fluency), metacognitive knowledge of tasks (difficult, high demands), and metacognitive knowledge of strategies (avoidance strategies) (as examined with a relative scale that has been used in young students) positively correlate with each other at different time points in aMCI patients or is there a cognitive disorganization as the disease progresses, not only as expected in cognitive performance, but also in metacognitive knowledge of the aMCI patients?; (b) Is there a deteriorating metacognitive knowledge pattern for the aMCI patients compared to healthy controls?; and (c) Are there brain volume changes correlating with metacognitive knowledge self-reports in the aMCI group?

## 2. Materials and Methods

The sample was derived from a previous study [16]. A total of 24 older adults (≥65 years) with a diagnosis of amnestic MCI (aMCI) without comorbid depression at baseline testing, who agreed to be followed with multiple brain magnetic resonance imaging (MRI) scans in Northern Greece, participated voluntarily in the study. The patients underwent a 3-Tesla (3-T) MRI and a detailed neuropsychological assessment during a 12-month period three times and did not receive any other intervention. Healthy controls (n = 24) were approached from a larger pool of participants [16], and one-to-one matching was followed, based on the demographics of the patient group (no statistically significant differences regarding age (t(28) = 0.090, *p* = 0.929), education years (t(28) = 0.102, *p* = 0.920), and identical gender frequency (see Table 1)). Healthy controls were examined with the detailed neuropsychological tests, but did not have to be tested and retested with 3-Ts. All participants completed the full neuropsychological testing, with no drop-off to report.

The neuropsychological assessment was simultaneous with the 3-Ts. The neuropsychological assessment included the following tests: Mini-Mental State Examination (MMSE) for overall cognition screening [27]; the Geriatric Depression Scale (GDS-15) in order to assess depression [28]; the Clinical Dementia Rating (CDR) for the investigation (through an interview with the caregiver) of the domains of cognitive and functional performance of the patient [29]; the Alzheimer’s Disease Assessment Scale (ADAS) for the examination of cognitive and non-cognitive symptoms [30]; the Neuropsychiatric Inventory (NPI) for the detection of psychiatric symptoms [31]; the Digit Cancellation Test [32]; the Rey Auditory Verbal Learning Test (RAVLT-immediate, delayed and recall conditions) which is used in order to evaluate verbal memory [33]; the Clock Drawing Test (CDT-immediate drawing and copy) as a measure of spatial dysfunction and neglect [34]; the Trail-Making Test Parts A and B, which focus on the assessment of visual attention and task switching [35]; the Wechsler Adult Intelligence Scale (WAIS-R) Digit Symbol which measures speed and memory [36]; the Boston Naming Test (BNT), which is used in order to assess naming-word retrieval [37]; the Digit Span Memory Test Forward Condition, which measures attention and the Digit Span Memory Backward Condition, which measures executive functioning [38]; and the Verbal Fluency Test (FAS; letter fluency and category fluency), which assesses lexical retrieval and production [39] (see [16] for the detailed scores of the abovementioned tests). There were no statistically significant differences regarding the mood of the two groups of participants as measured with GDS-15 at baseline as zero scores were reported by all members of the two groups, at six months (t(28) = 0.716, *p* = 0.480), and at twelve months (t(28) = 0.473, *p* = 0.640) (see Table 1).

Additionally, all participants completed the Metacognitive Knowledge in Mathematics Questionnaire (MKMQ), which was initially used in students. In this study, words such as fellow students were changed so that its use was more appropriate for older adults. MKMQ includes seven subscales, namely metacognitive knowledge of the self (easiness, fluency), metacognitive knowledge of the self (difficulty, lack of fluency), metacognitive knowledge of tasks (easy, low demands), metacognitive knowledge of tasks (difficult, high demands), metacognitive knowledge of strategies (cognitive/metacognitive strategies), metacognitive knowledge of strategies (competence/enhancing strategies), and metacognitive knowledge of strategies (avoidance strategies) (see [26] for the full questionnaire). For each of the items there is 5-point Likert scale from 1 = not at all true of me to 5 = absolutely true of me. The Cronbach’s internal consistency for this sample at baseline was high for all subscales: metacognitive knowledge of the self (easiness, fluency) (α = 0.87), metacognitive knowledge of the self (difficulty, lack of fluency) (α = 0.96), metacognitive knowledge of tasks (easy, low demands) (α = 0.93), metacognitive knowledge of tasks (difficult, high demands) (α = 0.91), metacognitive knowledge of strategies (cognitive/metacognitive strategies) (α = 0.93), metacognitive knowledge of strategies (competence/enhancing strategies) (α = 0.92), and metacognitive knowledge of strategies (avoidance strategies) (α = 0.89).

A volumetric MRI was performed using Statistical Parametric Mapping (SPM 12) at three time points regarding the following volumes: white matter, grey matter, cerebrospinal fluid, and sixteen additional brain areas, such as the right angular gyrus, the left angular gyrus, the right amygdale, the left amygdala, the right precuneus, the left precuneus, the right hippocampus, the left hippocampus, the right parahippocampal gyrus, the left parahippocampal gyrus, the right thalamus, the left thalamus, the right medial superior frontal cortex, the left medial superior frontal cortex, the right medial frontal cortex, and the left medial frontal cortex. All scans were straight–non oblique and the Human CORE Scan Protocol was followed (no adjustments were made to this protocol), Plane/Tri-Planar Scout/Calibration Scan, Sagittal 3D Accelerated MPRAGE/IRSPGR, Sagittal 3D FLAIR • Axial T2 Star/GRE.

## 3. Results

Statistical analyses were performed using SPSS software. The independent sample two-tailed t-tests used for comparing the performance of healthy controls and aMCI patients revealed (as expected) statistically significant differences for MMSE (t(28) = 4.400, *p* < 0.001) at time point 1; at time point 2 and at time point 3 differences were also found between the two groups for MMSE (t(28) = 2.718, *p* = 0.011 and t(28) = 3.833, *p* = 0.001, respectively).

At all three time points, a number of statistically significant Pearson correlations were found among the MKMQ subscales for the aMCI sample (see Table 2 for baseline testing, Table 3 for testing at six months and Table 4 for testing at twelve months).

Statistically significant differences were also found at the three time points for all subscales of MKMQ between the two groups. More specifically, at baseline: metacognitive knowledge of the self (easiness, fluency) (t(28) = 23.052, *p* < 0.001), metacognitive knowledge of the self (difficulty, lack of fluency) (t(28) = 13.946, *p* < 0.001), metacognitive knowledge of tasks (easy, low demands) (t(28) = 17.338, *p* < 0.001), metacognitive knowledge of tasks (difficult, high demands) (t(28) = 8.595, *p* < 0.001), metacognitive knowledge of strategies (cognitive/metacognitive strategies) (t(28) = 3.891, *p* = 0.001), metacognitive knowledge of strategies (competence/enhancing strategies) (t(28) = 8.189, *p* < 0.001), and metacognitive knowledge of strategies (avoidance strategies) (t(28) = 17.737, *p* < 0.001).

At six months, group differences were also found: metacognitive knowledge of the self (easiness, fluency) (t(28) = 23.846, *p* < 0.001), metacognitive knowledge of the self (difficulty, lack of fluency) (t(28) = 13.873, *p* < 0.001), metacognitive knowledge of tasks (easy, low demands) (t(28) = 18.552, *p* < 0.001), metacognitive knowledge of tasks (difficult, high demands) (t(28) = 8.774, *p* < 0.001), metacognitive knowledge of strategies (cognitive/metacognitive strategies) (t(28) = 3.986, *p* < 0.001), metacognitive knowledge of strategies (competence/enhancing strategies) (t(28) = 9.850, *p* < 0.001), and metacognitive knowledge of strategies (avoidance strategies) (t(28) = 18.767, *p* < 0.001).

At twelve months, group differences were also present: metacognitive knowledge of the self (easiness, fluency) (t(28) = 22.813, *p* < 0.001), metacognitive knowledge of the self (difficulty, lack of fluency) (t(28) = 13.123, *p* < 0.001), metacognitive knowledge of tasks (easy, low demands) (t(28) = 16.679, *p* < 0.001), metacognitive knowledge of tasks (difficult, high demands) (t(28) = 7.412, *p* < 0.001), metacognitive knowledge of strategies (cognitive/metacognitive strategies) (t(28) = 4.703, *p* < 0.001), metacognitive knowledge of strategies (competence/enhancing strategies) (t(28) = 9.259, *p* < 0.001), and metacognitive knowledge of strategies (avoidance strategies) (t(28) = 22.671, *p* < 0.001) (see Table 5).

In addition to that, Spearman’s rank correlation coefficients (Rho) were computed for all brain volumes and MKMQ subscales for the aMCI group. For the different brain area volumes, the relative values are presented as calculated from the formula absolute specific area (e.g., hippocampal) volume (mm^3^) divided by total brain volume (mm^3^). The choice to use Spearman’s rho was made due to the small sample size for the correlations between brain volumes and different aspects of metacognitive knowledge. Only two statistically significant positive correlations were found between knowledge of strategies (avoidance strategies) and right amygdala (rho = 0.752, *p* = 0.001) and knowledge of strategies (avoidance strategies) and left amygdala (rho = 0.662, *p* = 0.007) at baseline. At six months, again only knowledge of strategies (avoidance strategies) (among other metacognitive measures) was found to correlate in a statistically significant way with white matter volume (rho = −0.655, *p* = 0.008), and with the left amygdala volume (rho = 0.559, *p* = 0.030). At twelve months, metacognitive knowledge and use of avoidance strategies was found to correlate with the right parahippocampal (rho = −0.535, *p* = 0.040) and left parahippocampal volume (rho = −0.531, *p* = 0.042). Another point of interest is that the difference between the last volume measurement (at 12 months) from the first volume measurement (at baseline) indicated that there is only one statistically significant correlation between knowledge of strategies (avoidance strategies) and left amygdala volume (rho = 0.615, *p* = 0.019) (Table 6).

**Figure 1:**
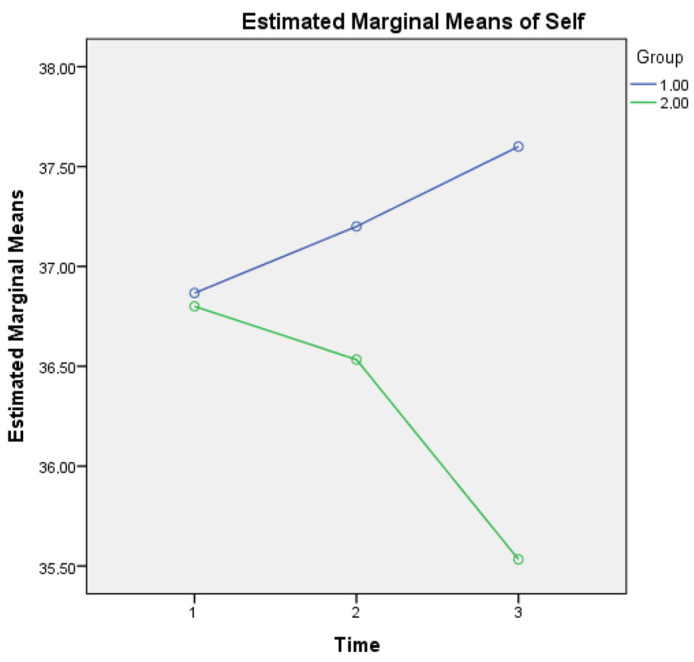
Metacognitive knowledge of self for the two groups in three time points. Note: Group 1 = Healthy; Group 2 = aMCI patients.

A two-way repeated measures ANOVA was also applied (one MANOVA, with the three (instead of seven) subscales as outcome or dependent variables, the three timepoints (within-subject) and the two groups (between-subject) as independent variables). For metacognitive knowledge of the self, a statistically significant interaction was found for Group × Time (F(2, 1733) = 3.132, *p* = 0.005) as it seems that healthy controls show a growing metacognitive knowledge of self in comparison to aMCI patients who present a diminishing relevant score (see Figure 1). This finding may be due to the declining cognitive capacities of aMCI patients, which may also affect their statements regarding metacognitive knowledge of self.

**Figure 2:**
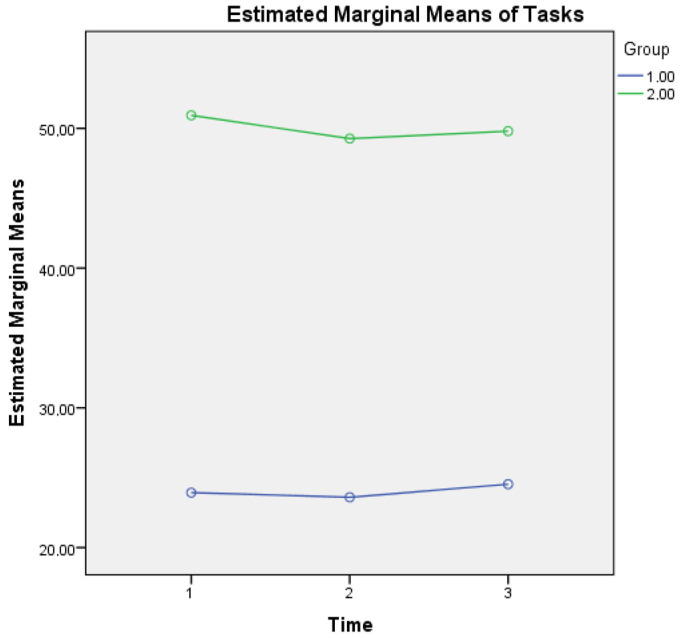
Metacognitive knowledge of tasks for the two groups in three time points. Note: Group 1 = Healthy; Group 2 = aMCI patients.

For metacognitive knowledge of tasks, a main effect of time was found (F(2, 1706) = 3.832, *p* = 0.028) as well as an interaction effect of Group × Time (F(2, 1706) = 2.943, *p* = 0.050), a finding that reveals that healthy controls report higher metacognitive knowledge of tasks (low levels of difficulty), based also on their better cognitive performance (as depicted in MMSE scores of overall cognitive performance at three time points) compared to aMCI patients. A possible explanation of the higher scores of reporting difficulty in metacognitive knowledge of tasks in aMCI patients could be based on the fact that changes in their overall cognition also influence their responses regarding perceived demands of mathematical operations that these individuals were taught in their early school years (see Figure 2).

**Figure 3:**
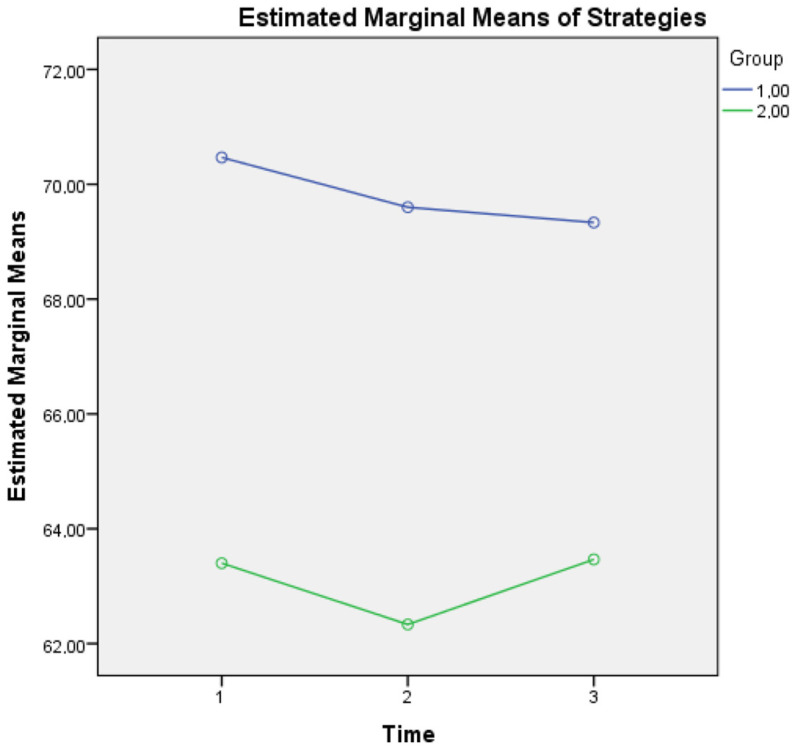
Metacognitive knowledge of strategies for the two groups in three time points. Note: Group 1 = Healthy; Group 2 = aMCI patients.

For metacognitive knowledge of strategies, an interaction effect of Group × Time (F(2, 1674) = 560, *p* = 0.020) was found, a finding that supports the idea that although healthy controls seem to present a diminishing tendency of using demanding metacognitive strategies most of the time, they still present a clear difference above the mean scores of aMCI patients at all three time points (see Figure 3).

## 4. Discussion

Differences between healthy controls and aMCI patients in all aspects of metacognitive knowledge (self, task, and strategies) is an interesting new finding that could assist clinicians in the preparation of relevant cognitive and metacognitive interventions for this diagnostic group. Overall, healthy controls reported higher metacognitive knowledge of the self (easiness, fluency) in contrast to aMCI patients, while more difficulty was experienced by aMCI patients regarding metacognitive knowledge of the self (difficulty, lack of fluency). Healthy controls find tasks with low demands less difficult (such as addition, subtraction etc.) than metacognitive knowledge of tasks (easy, low demands), as well as more difficult tasks with higher demands (metacognitive knowledge of tasks (difficult, high demands)) such as fractions, compared to aMCI patients. Metacognitive knowledge of strategies (cognitive/metacognitive strategies) and metacognitive knowledge of strategies (competence/enhancing strategies) are used more by healthy controls compared to aMCI patients, based on their self-reports. Finally, metacognitive knowledge of strategies (avoidance strategies) is mentioned more by aMCI patients compared to healthy older adults. Given that both groups had the same demographics, including education (as measured in years), these differences cannot be explained by educational level differences, but could be considered as symptoms revealed in aMCI.

It is of interest that when metacognitive knowledge of self was inserted as a composite variable, healthy controls showed higher metacognitive knowledge of self and metacognitive strategies in all three time points, but higher metacognitive knowledge of tasks and lower expressed perceived difficulty compared to aMCI patients. Although these findings are considered as expected, they are examined for the first time in this study. These findings demonstrate the relatively intact metacognitive control in healthy aging [40], but we cannot exclude that these statements may also depict overestimations on the part of the healthy controls or underestimations made by the aMCI patients, although the cognitive and metacognitive deficits of the aMCI diagnostic group could explain a possible inaccuracy of self-reports in aMCI [41].

Although the general scientific literature links metacognition to frontal structures, this study failed to detect direct significant relationships between frontal volumes in aMCI and metacognition, a finding that may be explained by the slight volumetric changes for this group of patients, compared with the more prominent neuronal death and therefore volume decrease found in AD patients. Another point that is of interest is that the larger the size of the amygdala (both left and right) in aMCI patients, the more the reported avoidance strategies. The amygdala is considered to be a limbic structure involved in emotion regulation, emotional learning and memory [42,43]. This may serve as an addition to existing findings that support so far that prolonged experienced stress leads to increases in measures of amygdala structure in animals, such as rodents [44,45,46]. This means that the amygdala is one of the few crucial structures that generally increases in volume in response to chronic stress [44,46,47]. In addition to that, parahippocampal volumes seem to be of interest, given that parahippocampal atrophy is linked to cognitive decline [48,49] and may be linked to the avoidance that individuals express when they are confronted by cognitive mathematical problems. The point regarding the parahippocampal volume correlation with metacognitive knowledge and use of avoidance strategies should be approached with caution. A plethora of studies supports reduction of hippocampus and parahippocampal structures due to chronic stress, not only in PTSD patients [50,51,52]. Although in this sample depressive symptomatology was examined before inclusion to the study protocol, and participants with high GDS-15 scores were excluded, no relevant tool examining stress levels was administered due to time restrictions in order to exclude possible chronic-stress-induced volume loss.

The above could be used in future clinical practice, and assist in the diagnosis of possible deficits in the everyday lives of aMCI patients, especially when arithmetic, mathematical, and financial problems are encountered. In addition to that, the fact that only avoidance strategies have moderate correlations with brain measurements should be investigated in further detail in future research.

One of the major limitations of this study is the small size, but this is a problem that was solved through one-to-one matching of the two groups of participants. In addition to that, the comparisons between the two groups lack support from MRI data for the HC group, thus this study was based on a self-report questionnaire; however, given the literature on the nature of metacognition [47], this is the most useful and recommended way of approaching (through self-perception statements) metacognitive knowledge. Future studies should examine in more detail the detected brain areas that correlate with metacognitive self-reported changes not only in aMCI patients, but also in other groups of patients suffering from mild as well as major neurocognitive disorders due to different etiologies.

## 5. Conclusions

These findings indicate that even at baseline, there are some so far disregarded interesting relationships between metacognitive easiness, which is negatively correlated with metacognitive knowledge of tasks with low demands, thus implying that aMCI patients claim that they can easily solve mathematical problems and at the same time they find low demand tasks very difficult, such as those that include fundamental operations in mathematics (e.g., addition and subtraction). Despite this contradiction, aMCI patients show overall higher perceived difficulty in metacognitive knowledge of tasks, and lower metacognitive knowledge of self and metacognitive strategies compared to healthy controls. The study results are of utmost importance for clinical practice and everyday life, as altered metacognitive self-evaluations regarding mathematical tasks may render older adults vulnerable to financial abuse.

## Figures and Tables

**Table 1:** Means and SDs for demographics, MMSE and GDS at three time points for aMCI patients and healthy controls (HC).

Demographic Variables and Neuropsychological Tests	Diagnostic Group	Time 1 Baseline	Time 2 Six Months	Time 3 Twelve Months
Age (years)	aMCI	70.0 (8.31)		
	HCs	70.26 (7.90)		
Education (years)	aMCI	10.00 (3.70)		
	HCs	10.13 (3.48)		
Sex (%female)	aMCI	66.7%		
	HCs	66.7%		
MMSE	aMCI	28.13 (1.40)	28.40 (1.84)	28.00 (1.69)
	HCs	29.80 (0.14)	29.73 (0.45)	29.73 (0.43)
GDS-15	aMCI	0 (0.00)	1.46 (1.55)	1.73 (1.86)
	HCs	0 (0.00)	1.33 (0.91)	1.46 (1.12)

**Table 2:** Baseline correlations among MKMQ subscales.

Subdomains of MKMQ	MKMQ Easiness	MKMQ Difficulty	MKMQ Low Demands	MKMQ High Demands	MKMQ Cognitive/Metacognitive Strategies	MKMQ Enhancing Strategies	MKMQ Avoidance
MKMQ Easiness	1	−0.925 **	−0.932 **	−0.876 **	0.594 **	0.866 **	−0.925 **
		0.000	0.000	0.000	0.001	0.000	0.000
MKMQ Difficulty	−0.925 **	1	0.929 **	0.850 **	−0.614 **	−0.831 **	0.924 **
	0.000		0.000	0.000	0.000	0.000	0.000
MKMQ Low demands	−0.932 **	0.929 **	1	0.845 **	−0.593 **	−0.770 **	0.929 **
	0.000	0.000		0.000	0.001	0.000	0.000
MKMQ High demands	−0.876 **	0.850 **	0.845 **	1	−0.578 **	−0.743 **	0.820 **
	0.000	0.000	0.000		0.001	0.000	0.000
MKMQ Cognitive/Metacognitive strategies	0.594 **	−0.614 **	−0.593 **	−0.578 **	1	0.397 *	−0.543 **
	0.001	0.000	0.001	0.001		0.030	0.002
MKMQ Enhancing strategies	0.866 **	−0.831 **	−0.770 **	−0.743 **	0.397 *	1	−0.789 **
	0.000	0.000	0.000	0.000	0.030		0.000
MKMQ Avoidance	−0.925 **	0.924 **	0.929 **	0.820 **	−0.543 **	−0.789 **	1
	0.000	0.000	0.000	0.000	0.002	0.000	

** Correlation is significant at the 0.01 level (2-tailed). * Correlation is significant at the 0.05 level (2-tailed).

**Table 3:** Correlations at six months among MKMQ subscales.

Subdomains of MKMQ	MKMQ Easiness	MKMQ Difficulty	MKMQ Low Demands	MKMQ High Demands	MKMQ Cognitive/Metacognitive Strategies	MKMQ Enhancing Strategies	MKMQ Avoidance
MKMQ Easiness	1	−0.915 **	−0.935 **	−0.868 **	0.624 **	0.883 **	−0.925 **
		0.000	0.000	0.000	0.000	0.000	0.000
MKMQ Difficulty	−0.915 **	1	0.926 **	0.860 **	−0.641 **	−0.845 **	0.909 **
	0.000		0.000	0.000	0.000	0.000	0.000
MKMQ Low demands	−0.935 **	0.926 **	1	0.868 **	−0.624 **	−0.799 **	0.938 **
	0.000	0.000		0.000	0.000	0.000	0.000
MKMQ High demands	−0.868 **	0.860 **	0.868 **	1	−0.597 **	−0.752 **	0.819 **
	0.000	0.000	0.000		0.000	0.000	0.000
MKMQ Cognitive/Metacognitive strategies	0.624 **	−0.641 **	−0.624 **	−0.597 **	1	0.427 *	−0.561 **
	0.000	0.000	0.000	0.000		0.019	0.001
MKMQ Enhancing strategies	0.883 **	−0.845 **	−0.799 **	−0.752 **	0.427 *	1	−0.844 **
	0.000	0.000	0.000	0.000	0.019		0.000
MKMQ Avoidance	−0.925 **	0.909 **	0.938 **	0.819 **	−0.561 **	−0.844 **	1
	0.000	0.000	0.000	0.000	0.001	0.000	

** Correlation is significant at the 0.01 level (2-tailed). * Correlation is significant at the 0.05 level (2-tailed).

**Table 4:** Correlations at twelve months among MKMQ subscales.

Subdomains of MKMQ	MKMQ Easiness	MKMQ Difficulty	MKMQ Low Demands	MKMQ High Demands	MKMQ Cognitive/Metacognitive Strategies	MKMQ Enhancing Strategies	MKMQ Avoidance
MKMQ Easiness	1	−0.924 **	−0.930 **	−0.843 **	0.678 **	0.878 **	0.942 **
		0.000	0.000	0.000	0.000	0.000	0.000
MKMQ Difficulty	−0.924 **	1	0.877 **	0.804 **	−0.714 **	−0.862 **	0.905 **
	0.000		0.000	0.000	0.000	0.000	0.000
MKMQ Low demands	−0.930 **	0.877 **	1	0.834 **	−0.681 **	−0.792 **	0.933 **
	0.000	0.000		0.000	0.000	0.000	0.000
MKMQ High demands	−0.843 **	0.804 **	0.834 **	1	−0.614 **	−0.715 **	0.774 **
	0.000	0.000	0.000		0.000	0.000	0.000
MKMQ Cognitive/Metacognitive strategies	0.678 **	−0.714 **	−0.681 **	−0.614 **	1	0.535 **	−0.620 **
	0.000	0.000	0.000	0.000		0.002	0.000
MKMQ Enhancing strategies	0.878 **	−0.862 **	−0.792 **	−0.715 **	0.535 **	1	−0.840 **
	0.000	0.000	0.000	0.000	0.002		0.000
MKMQ Avoidance	−0.942 **	0.905 **	0.933 **	0.774 **	−0.620 **	−0.840 **	1
	0.000	0.000	0.000	0.000	0.000	0.000	

** Correlation is significant at the 0.01 level (2-tailed).

**Table 5:** Means and SDs for MKMQ subscales at three time points for aMCI patients and healthy controls (HC).

MKMQ	Diagnostic Group	Time 1 Baseline	Time 2 Six Months	Time 3 Twelve Months
Metacognitive knowledge of the self (easiness, fluency)	aMCI	7.26 (1.62)	7.46 (1.95)	7.40 (1.72)
	HCs	26.13 (2.70)	25.80 (2.24)	26.00 (2.64)
Metacognitive knowledge of the self (difficulty, lack of fluency)	aMCI	29.54 (4.30)	29.06 (3.82)	28.13 (3.13)
	HCs	10.73 (2.93)	11.40 (3.11)	11.60 (3.01)
Metacognitive knowledge of tasks (easy, low demands)	aMCI	27.40 (1.63)	26.80 (1.69)	27.20 (1.74)
	HCs	11.80 (3.01)	11.66 (2.62)	12.00 (3.07)
Metacognitive knowledge of tasks (difficult, high demands)	aMCI	23.53 (1.50)	22.46 (1.76)	22.60 (1.98)
	HCs	12.13 (4.91)	11.93 (4.30)	12.53 (4.98)
Metacognitive knowledge of strategies (cognitive/metacognitive strategies)	aMCI	27.53 (10.31)	27.46 (8.36)	28.13 (6.88)
	HCs	38.66 (4.04)	37.53 (5.06)	37.86 (4.10)
Metacognitive knowledge of strategies (competence/enhancing strategies)	aMCI	10.20 (3.36)	9.60 (3.31)	10.33 (2.52)
	HCs	21.36 (4.25)	22.33 (2.35)	21.66 (4.01)
Metacognitive knowledge of strategies (avoidance strategies)	aMCI	25.66 (2.19)	25.26 (1.94)	25.00 (1.13)
	HCs	10.13 (2.58)	9.73 (2.54)	9.80 (2.33)

**Table 6:** Brain volumes according to MRI scans at three time points for aMCI patients.

Brain Volumes	Time 1		Time 2		Time 3	
	Mean	SD	Mean	SD	Mean	SD
Grey matter	615.5000	59.26440	604.4000	65.88930	602.0000	60.20350
White matter	483.7143	32.52083	478.3333	40.77055	472.5000	46.50352
Cerebrospinal fluid	249.0000	31.49115	244.6000	31.38198	243.7143	31.70156
Left amygdala	1.1146	0.15972	1.0783	0.16109	1.1033	0.15351
Right amygdala	1.0668	0.13987	1.0692	0.13678	1.0665	0.15446
Left angular gyrus	5.9858	1.20545	5.8498	1.20393	5.8760	1.09782
Right angular gyrus	7.4571	1.17819	7.1997	1.20355	7.0855	1.14347
Left frontal medial cortex	1.3699	0.18273	1.3324	0.19985	1.2892	0.24053
Right frontal medial cortex	1.1333	0.16789	1.1132	0.20153	1.0337	0.24338
Left superior medial frontal gyrus	4.4414	0.49929	4.2333	0.58564	4.1997	0.61565
Right superior medial frontal gyrus	4.4362	0.60162	4.3891	0.56302	4.3935	0.56785
Left hippocampus	3.8314	0.25130	3.7617	0.28969	3.7528	0.36514
Right hippocampus	4.1967	0.19877	4.1481	0.30571	4.1417	0.29808
Left precuneus	8.0972	0.91730	78278	1.10038	7.6520	1.17142
Right precuneus	8.7092	0.91615	8.4832	1.12564	8.4140	1.00827
Left parahippompal gyrus	2.4525	0.18433	2.4438	0.20976	2.4340	0.23914
Right parahippocampal gyrus	2.2660	0.22390	2.2555	0.21726	2.2477	0.25184
Left thalamus	4.6322	0.67738	4.5259	0.60639	4.4768	0.67836
Right thalamus	4.5693	0.86450	4.4524	0.71702	4.3926	0.82417

## Data Availability

The data presented in this study are available on request from the corresponding author. The data are not publicly available due to privacy issues.
